# Alfaisal University's Academic Success Center: An Individualized Peer-Assisted Learning Program for Mutual Tutor-Student Advancement

**DOI:** 10.7759/cureus.44883

**Published:** 2023-09-08

**Authors:** Hamna Abdul Muthalib, Faaezuddin Syed, Tehreemah Raziq, Emily M Wilson, Muhammad Raihan Sajid

**Affiliations:** 1 Medicine, Alfaisal University College of Medicine, Riyadh, SAU; 2 English, Alfaisal University, Riyadh, SAU; 3 Pathology, Alfaisal University College of Medicine, Riyadh, SAU

**Keywords:** peer tutor, academic success center, peer mentoring, student well-being, peer assisted learning

## Abstract

Introduction: Peer-assisted learning (PAL) is an educational strategy whereby students teach other students. PAL presents many advantages to the institution, tutors, and tutees. It can benefit the university by presenting a cost-effective approach where the efforts and time of faculty are reduced. We describe a retrospective analysis detailing the structure, function, and effectiveness of the Academic Success Center (ASC) at Alfaisal University, Riyadh, Saudi Arabia, since 2020. The ASC encompasses various types of PAL methods. It is the only PAL program in Saudi Arabia whereby its tutors, referred to as student consultants, are compensated financially.

Methods: We retrospectively analyzed the total number of appointments, the total number of students who accessed the center, as well as the breakdown between different colleges and academic years, and their satisfaction level with the PAL mode of teaching.

Results: Our results indicate a high level of appointments for these PAL sessions increasing on a yearly basis and a high level of satisfaction from both the tutors and the tutees.

Conclusion: PAL is an effective teaching and mentoring modality with high retention rates that has contributed to an increased sense of well-being among students who have utilized these services.

## Introduction

Peer-assisted learning (PAL) is an educational strategy whereby students (tutors) teach other students (tutees) [1]. According to the literature, PAL has been categorized into peer learning (both the tutor and tutee belong to the same class or academic level) and near-peer learning (when tutors are senior to their tutees). Its sub-classification establishes six terms based on the tutor-to-tutee ratio: mentoring is for a ratio of 1:1 or 1:2, tutoring is for a ratio of 1:3-10, and didactic is of the ratio 1:<10 [1]. Therefore, PAL is also known as peer mentoring and near-peer mentoring in the literature. 

Most studies have found that PAL is an effective educational and mentoring tool. One study compared pre-test and post-test evaluations after using PAL in a pediatrics course and showed that there was a 24% improvement in post-test scores [2]. A meta-analysis demonstrated that PAL can be an effective teaching tool for those studying medicine. Medical students' learning experiences may be improved by educational interventions in courses that incorporate PAL methods [3]. 

PAL presents many advantages to the institution, tutors, and tutees. It can benefit the university by presenting a cost-effective approach that reduces the time and effort of faculty. From the perspective of peer tutors, extrinsic benefits (monetary reward) and intrinsic benefits have been recognized, with studies showing that intrinsic benefits overshadow financial rewards [4]. Some of the intrinsic benefits of PAL include leadership, coaching, learning skill development, confidence and intrinsic motivation enhancement, and the possibility of fostering an interest in academic careers [4]. The benefits of PAL to tutees are more emphasized in literature and include encouragement of critical thinking via exploration and argument as well as problem-solving skills [3]. The PAL environment has also been described as a safe space, especially for first-year university students, because it has been shown to alleviate student anxiety and enhance well-being [5]. Moreover, many medical councils in countries such as the US, UK, Germany, and Canada have labeled PAL a requirement or have recommended it in the medical curriculum, emphasizing its importance, particularly in skill-based labs [6]. To enforce standards and ensure the quality of teaching, peer tutors need to be trained and their sessions monitored regularly. One such study in Germany highlighted training methods used for peer tutors who teach both across colleges in the university and across disciplines with the training focused on communication, self-reflection, and group management [7].

The Academic Success Center (ASC) at Alfaisal University was established in September 2020 and encompasses various types of PAL methods as listed above. It is the only PAL program in Saudi Arabia whereby its tutors, referred to as student consultants, are compensated financially at an hourly rate. Student consultants attend regular training sessions and workshops held at the ASC to improve their teaching skills. Furthermore, the ASC’s director and designated teaching assistant also observe sessions once per semester and then meet with tutors for reflection sessions to improve their practice. Appointment schedules are flexible and allow consultants to consider their own classes and exam schedules. The minimum and maximum appointment hours are 4 to 152 per week. Student consultants are also encouraged to host workshops addressing time management, study methods, handling stress as well as balancing studies and social life. These opportunities grant them the confidence and exposure to public speaking as well as handling a large in front of large audiences.

The ASC covers a variety of college courses including English, Physics, Biostatistics, Mathematics, accounting, and undergraduate medical courses and modules, with sessions being either in-person or online and lasting from 30 minutes to an hour. Students may book a maximum of 4 appointments per week and are encouraged to send study materials ahead of time to facilitate smooth delivery of the session.

As there is a paucity of studies describing the effectiveness of PAL in the Gulf and middle eastern region, this retrospective descriptive analysis was performed to address the gap in literature from this part of the world and to assess the satisfaction of students with the PAL support services at Alfaisal University, Riyadh, Saudi Arabia.

## Materials and methods

This is a retrospective descriptive study that aims to describe the structure, function, and effectiveness of the ASC at Alfaisal University, Riyadh, Saudi Arabia, since 2020.

The ASC uses the WCONLINE 8.1.5 software version 2.3.3 which is used to schedule appointments and generate data via client reports and feedback forms. After a session, a client report is filled out by the consultant (peer tutor), and a feedback form is sent to students to assess their interaction with the consultant. The feedback survey is voluntary and does not impact future appointments, as the student’s identity is not disclosed to the tutors. The survey includes tutee demographics, the consultant they booked with, the subject discussed, an open-ended question regarding comments and suggestions, and three questions asking the tutee; to rate their student consultant as excellent/very good/okay/poor, their willingness to return to the ASC as yes/maybe/no, and their willingness to recommend the ASC to a peer as yes/maybe/no. 

This data is used to retrospectively analyze the total number of appointments, the total number of students who accessed the ASC, as well as the breakdown between different colleges and academic years. The mean and median for each parameter were calculated and analyzed via Microsoft Excel. 

The inclusion criteria for this study were all appointments made from September 5, 2020, to January 31, 2023. Data from appointments that were canceled or marked as no-shows were excluded. 

## Results

**Figure 1:**
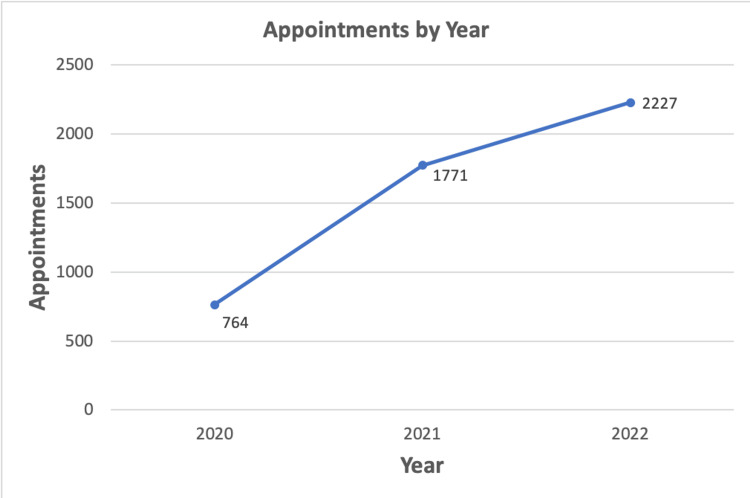
Showing the growth of ASC with an increasing number of appointments ASC: Academic Success Center

A total of 4762 appointments took place at the ASC of Alfaisal University in the period 2020 to 2023. The total number of appointments in the year 2020-2021 went up to 764. In the academic year 2021-2022, 1771 appointments were held, and the number of appointments in the 2022-2023 period increased to 2227 (Figure 1). Female students made up a larger proportion of the overall appointments with an approximate ratio of 3:1. A total of 921 female students made recurring visits to the ASC with a tally of 3339 appointments, constituting 70% of the sessions. Five hundred and sixty male students made continuous scheduled visits to the ASC with 1423 appointments.

**Figure 2:**
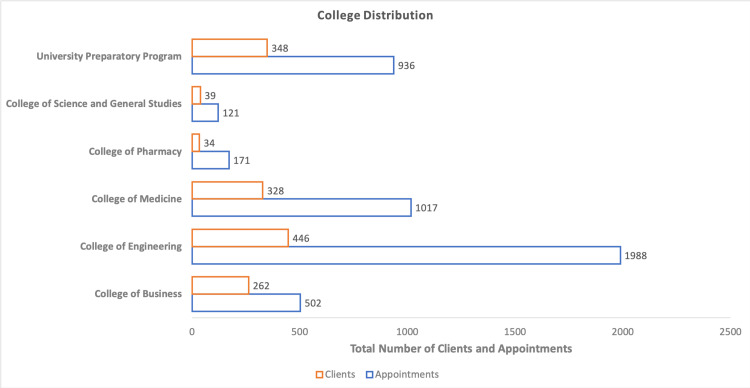
Showing the utilization of ASC by students of different colleges ASC: Academic Success Center

To provide the best service to students coming from a wide range of academic backgrounds, the ASC’s director recruited student consultants (peer tutors) with an interest in different fields. Fourteen versatile consultants were recruited in the year 2020-2021. As with the growing number of appointments, the demand for consultants was on the rise as well. An additional 10 consultants were recruited in each subsequent year increasing the total number of consultants from 14 to 24 in 2021-2022, and 24 to 34 in 2022-2023. A total of 1988 sessions were booked with the consultants by 446 clients. The highest number of active sessions were recorded in the field of Engineering, constituting approximately 42% of the total number of appointments. The College of Medicine accounted for 30% of all appointments, just behind Engineering. Next on the list were students from the University Preparatory Program and College of Business booking a total of 936 and 502 appointments by 348 and 262 clients respectively (Figure 2). 

By sorting the data according to their standing in college years, 4057 appointments representing 85% of the total sessions were booked by students in the freshman year (0-30 credit hours). The rest of the years including sophomore (31-60 credit hours), junior (61-90 credit hours), and senior (91-120 credit hours) comprised only 705 appointments.

The success of the ASC was echoed through the persistent return of the clients and high satisfaction levels from the students. The consultants received excellent feedback with 93% of the clients rating their consultants as excellent. Approximately 90% of the students displayed a desire to return to the ASC, with only 10% of the clients skeptical about returning. Of the users, 97% showed positive intent in recommending the ASC to their peers and colleagues. The satisfaction level of consultants was more than 90% as reported by the tutees. Approximately 90% of the students indicated their intention to return for these PAL sessions and almost 97% indicated that they would recommend the services of the ASC to other students.

## Discussion

This retrospective analysis of the performance of the PAL program at the ASC at Alfaisal University indicates a strong performance, acceptance, and utilization of the PAL services by the students across all colleges at the university. Most of the students attending the PAL sessions indicated high levels of satisfaction with the quality of teaching provided by the PAL tutors/consultants. Similar results are reported by other published studies, which emphasize an enhanced feeling of wellness and increased confidence among students utilizing PAL services [8].

An increase in the number of appointments was noted in this study, which demonstrates an increasing awareness of the utility of PAL services by the students at large, and similar results are reported by other studies [9]. The psychosocial role of mentoring PAL coaching has been linked to the personal and professional growth of mentees. It aids the mentees in learning the ropes and gets them ready for promotion within their institute [9]. Social interaction is the primary way that behaviors are learned. It has been hypothesized that the mentoring process fosters in peer mentors a dedication to professional progress as they support their peers, which leads to the development of transferrable abilities and qualities [8,9]. This has also been discussed in other studies that highlight the importance of tutees regarding PAL environments as a safe learning space [10,11]. Additionally, a paper published by Weyrich et al. discovered that students preferred PAL over faculty in certain settings and suggested that faculty be appointed as supervisors of these training sessions instead, for quality assurance purposes [12].

Most PAL programs around the world are centered on medical education and allied health science programs. For instance, a letter to the editor evaluated a group of students and concluded that they gained more confidence in performing clinical skills compared to a session conducted by faculty [12,13]. A similar study discovered that first-year medical students experienced less severe physical and emotional reactions on the first day of anatomy dissection when paired with third-year medical students [14]. PAL also positively impacts students’ exam performance and preparedness as proven by Batchelder et al. in a study [15]. After following a cohort of students for 4 years, they recorded statistically significant increases in exam preparedness (p = 0.001) and familiarity with exam-style questions (p = 0.004).

Several causes could account for the increased utilization of PAL sessions by students, including the fact that student tutors may have a deeper grasp and stronger retention of topics they prepared for and presented, while student learners are frequently more engaged in active learning and are comfortable to ask questions and clarify understanding with fellow students [16,17]. Additionally, PAL naturally promotes student ownership of their education and the development of vital skills for lifelong learning. This is even more crucial in light of the fact that physicians are essentially teachers/educators to both junior doctors and their patients. Furthermore, PAL provides the tutor with the skill and professionalism often seen missing in residency training [10]. From the perspective of the educator, faculty time is a finite resource that may be the rate-limiting step in offering medical students interactive learning experiences. PAL provides a method of teaching that emphasizes active learning while also making effective use of faculty time [9,16,17]. We believe that these reasons as reported in the literature were similarly reflected in our results.

## Conclusions

The retrospective analysis of the PAL program at Alfaisal University's Academic Success Center shows strong performance, acceptance, and utilization of PAL services by students across all colleges. The students attending PAL sessions expressed high levels of satisfaction with the quality of teaching provided by PAL tutors/consultants. The increase in the number of appointments indicates a growing awareness of PAL services' utility among students. Similar to other studies, the mentoring/coaching role of PAL has been linked to personal and professional growth for mentees. PAL environments provide safe learning spaces and foster transferrable abilities and qualities in peer mentors. The PAL program positively impacts students' exam performance and preparedness. PAL's effectiveness is attributed to student tutors' deeper grasp and stronger retention of topics, active learning engagement, and student ownership of education. Moreover, PAL offers an interactive teaching method that optimizes faculty time. These findings align with existing literature on PAL's benefits in medical education and allied health science programs.
